# Association between hemoglobin glycation index and all-cause mortality in patients with non–ST-segment elevation myocardial infarction undergoing percutaneous coronary intervention

**DOI:** 10.3389/fendo.2026.1847325

**Published:** 2026-05-25

**Authors:** Haodong Jiang, Yanlong Zhao, Yuanyuan Zhao, Shuai Wang, Wenliang Zhai, Zhi Liu

**Affiliations:** Department of Emergency, Xuanwu Hospital, Capital Medical University, Beijing, China

**Keywords:** all-cause mortality, hemoglobin, hemoglobin glycation index, non-ST-segment elevation myocardial infarction, percutaneous coronary intervention

## Abstract

**Background:**

The hemoglobin glycation index (HGI) has emerged as a potential marker for cardiovascular risk stratification. However, its prognostic value in patients with non-ST-segment elevation myocardial infarction (NSTEMI) undergoing percutaneous coronary intervention (PCI) remains unclear. This study investigated the association between HGI and all-cause mortality in this population.

**Methods:**

This single-center cohort study included 635 patients with NSTEMI who underwent PCI between January 2016 and July 2023. The primary endpoint was all-cause mortality. Kaplan–Meier analysis, Cox regression, restricted cubic spline (RCS) analysis, and subgroup analyses were performed.

**Results:**

Over a median follow-up of 60 months, higher HGI was associated with more favorable survival. In the fully adjusted model, a 1-unit increase in HGI was associated with a 43% lower risk of all-cause mortality (HR=0.57, 95% CI: 0.40–0.82, P=0.002). Patients in the highest HGI tertile also had a lower risk of mortality than those in the lowest HGI tertile (HR=0.26, 95% CI: 0.10–0.66, P=0.004). RCS analysis indicated a linear inverse association. Exploratory subgroup analyses suggested that the inverse association appeared more evident in men than in women.

**Conclusion:**

HGI was associated with all-cause mortality and may serve as a complementary marker for risk stratification in patients with NSTEMI undergoing PCI.

## 1 Introduction

Acute myocardial infarction (AMI) represents one of the most severe manifestations of cardiovascular disease and remains a leading cause of death worldwide (1). Non-ST-segment elevation myocardial infarction (NSTEMI) accounts for more than 60% of AMI cases and has received increasing clinical attention in recent years (2). Previous studies have reported that patients with NSTEMI remain at considerable long-term risk, with the 5-year incidence of all-cause mortality exceeding 6% even among those undergoing percutaneous coronary intervention (PCI) (3, 4). Therefore, identifying risk factors that can predict mortality in patients with NSTEMI is of considerable clinical importance.

Abnormal glucose metabolism is increasingly recognized as an important prognostic determinant in NSTEMI. Dysglycemia may worsen cardiovascular outcomes through multiple mechanisms, including acceleration of atherosclerotic progression, promotion of plaque instability, and exacerbation of myocardial injury (5). Current guidelines emphasize the importance of glycemic management in coronary artery disease and recommend maintaining glycated hemoglobin (HbA1c) within an appropriate target range, generally below 7% (6). However, although HbA1c is widely used as an indicator of long-term glycemic exposure, its value may be influenced by erythrocyte lifespan, genetic background, and interindividual variability in glycation, which may limit its ability to fully reflect overall glycemic status (7). Notably, the ACCORD trial showed that an intensive glucose-lowering strategy targeting uniformly lower HbA1c levels (<6%) failed to yield the anticipated benefit and instead increased mortality risk in patients with diabetes (8). These findings suggest that, although HbA1c remains clinically important, complementary markers may help provide a more comprehensive assessment of glycemic status and risk.

In recent years, increasing attention has been directed toward the application of novel metabolic and biochemical markers in cardiovascular risk stratification and prognostic assessment (9). The hemoglobin glycation index (HGI) is a novel glycemic marker defined as the difference between observed HbA1c and glucose-predicted HbA1c (10). It was initially proposed to identify patients with diabetes who may benefit from intensive glucose-lowering therapy (8). Subsequent studies have shown that HGI also has clinical relevance in primary cardiovascular prevention. For example, Ahn et al. reported that higher HGI was independently associated with an increased risk of cardiovascular disease among individuals with impaired glucose regulation (11). In addition, Jia et al. reported a close association between HGI and the severity of coronary lesions (12). Similarly, Evidence from heart failure populations has indicated that higher HGI is also associated with an increased risk of in-hospital clinical deterioration (13). Collectively, these findings suggest that HGI may serve not only as a marker of abnormal glucose metabolism but also as a potentially useful indicator of cardiovascular risk and prognosis.

Building on these findings, the prognostic value of HGI in patients with AMI has also attracted increasing attention. Growing evidence suggests that HGI is associated with all-cause mortality in patients with AMI (14–16). However, most available evidence has been derived from heterogeneous AMI populations or from critically ill patients requiring intensive care. Data specifically focusing on patients with NSTEMI undergoing PCI, a relatively homogeneous and clinically prevalent population, remain limited. Therefore, we sought to determine whether HGI was associated with all-cause mortality among patients with NSTEMI undergoing PCI, thereby providing further evidence regarding its prognostic relevance in this population.

## 2 Methods

### 2.1 Population

**Figure 1:**
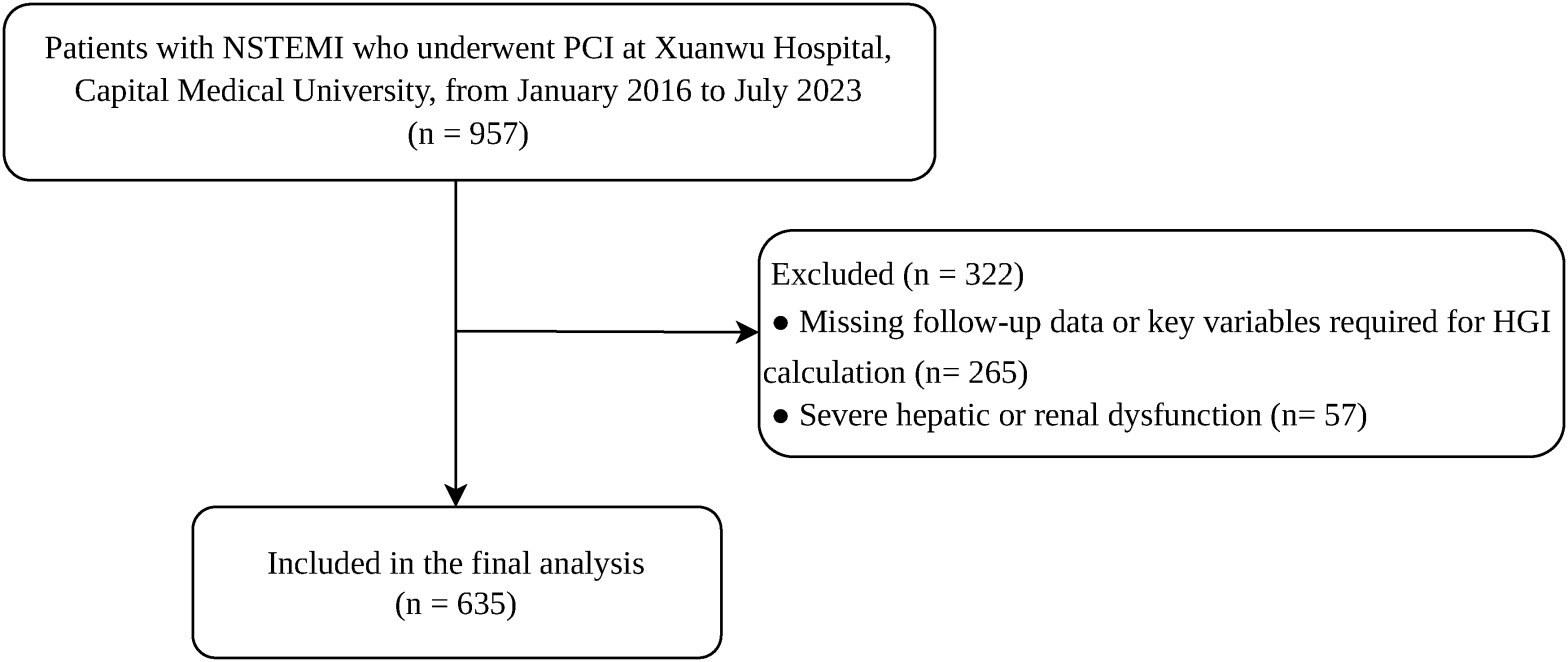
Flowchart of the study population selection.

This single-center cohort study included consecutive patients with NSTEMI who underwent PCI at Xuanwu Hospital, Capital Medical University, between January 2016 and July 2023. Patients were excluded if they had missing follow-up records, incomplete variables required for HGI calculation, significant hepatic or renal impairment, concomitant malignancy likely to affect survival, or previous coronary artery bypass grafting (CABG). After the exclusion process, 635 patients remained eligible for the final analysis (**Figure 1**). The study protocol was approved by the Ethics Committee of Xuanwu Hospital, Capital Medical University. The study was conducted in accordance with the Declaration of Helsinki.

### 2.2 Data collection and definition

**Figure 2:**
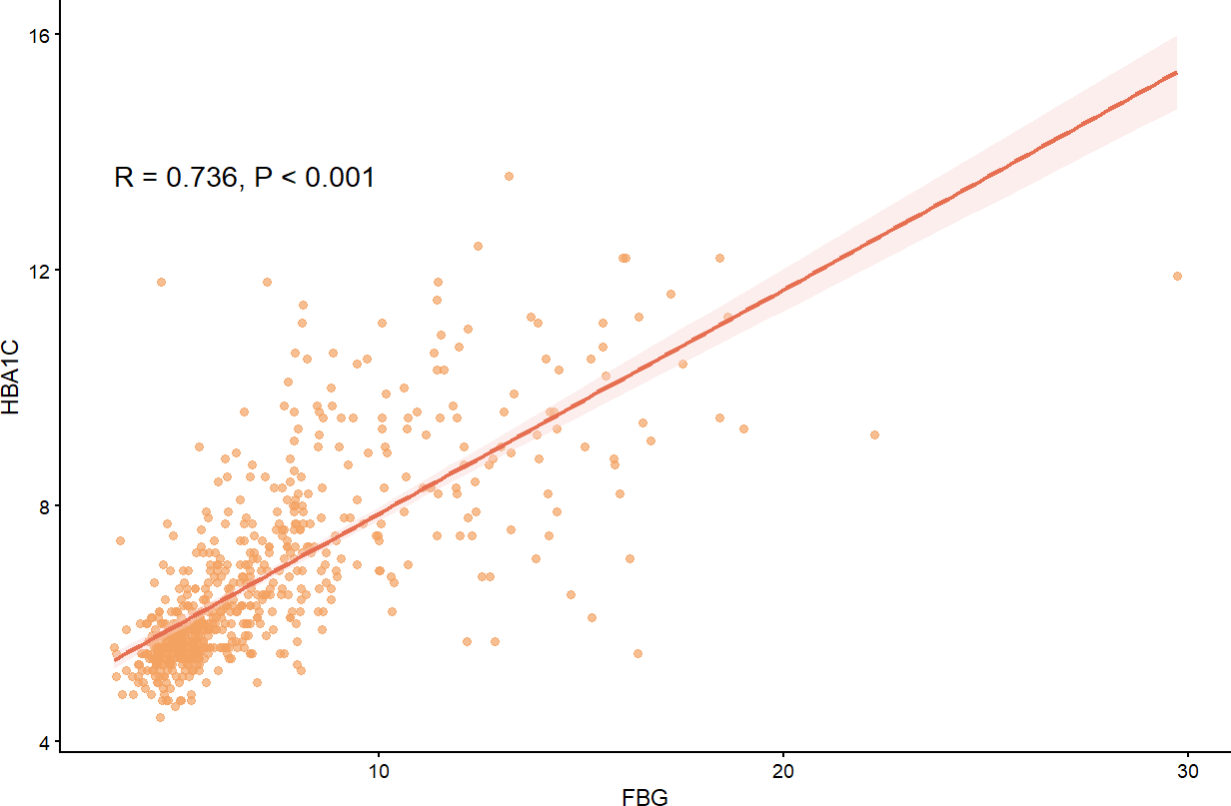
Linear regression analysis of FBG and HbA1c for HGI calculation. FBG, fasting blood glucose; HbA1c, glycated hemoglobin; HGI, hemoglobin glycation index.

Clinical characteristics were obtained from the institutional electronic database, including sex, age, BMI, smoking and drinking status, histories of hypertension and diabetes, previous PCI, Killip classification, and discharge medications. Blood samples were drawn after fasting overnight for more than 8h. Biochemical indices were measured in the central laboratory of Xuanwu Hospital, Capital Medical University, using established laboratory protocols, including hepatic and renal function indices, fasting blood glucose (FBG), serum lipid measurements, and HbA1c. Coronary angiograms were interpreted by at least two senior cardiologists, and lesion-related variables were determined by consensus. These variables included left main (LM) coronary artery disease and three-vessel disease. Diabetes was considered present if any of the following criteria were met: (1) a previous diagnosis of diabetes with ongoing glucose-lowering treatment; (2) FBG ≥ 7.0 mmol/L and/or 2-hour plasma glucose ≥11.1 mmol/L during an oral glucose tolerance test; (3) HbA1c ≥ 6.5%; (4) classic manifestations of hyperglycemia accompanied by a casual blood glucose concentration ≥11.1 mmol/L (17). A diagnosis of hypertension was assigned to patients with systolic blood pressure (SBP) ≥ 140 mmHg, diastolic blood pressure (DBP) ≥ 90 mmHg, or ongoing antihypertensive therapy (18). NSTEMI was identified in patients presenting with evidence of myocardial ischemia together with elevated cardiac troponin (cTn), in the absence of ST-segment elevation (19). HGI was calculated using the regression-based method originally proposed by Hempe et al. (10). Specifically, a regression equation describing the relationship between FBG and HbA1c was established within the study population to estimate predicted HbA1c, and HGI was defined as the difference between measured HbA1c and predicted HbA1c. Following this methodological framework, we constructed a regression model based on the relationship between admission FBG and HbA1c within our institutional cohort of patients with NSTEMI undergoing PCI. The resulting equation was: predicted HbA1c = 0.379 × FBG+4.0671 (**Figure 2**).

### 2.3 Interventions and management

PCI and periprocedural care were performed according to contemporary guideline recommendations (20). Decisions regarding the specific interventional strategy were made by experienced interventional cardiologists on an individual basis.

### 2.4 Follow-up and endpoint

All participants received guideline-recommended medical therapy at discharge, unless clear contraindications were present or the treatment was not tolerated (20). Long-term follow-up was conducted through April 2025. Follow-up information was obtained by trained investigators through telephone interviews, outpatient visits, and review of the electronic medical records, with supplementary confirmation from family members when necessary. The outcome was all-cause mortality. The median follow-up time was 60 months (IQR: 38–85 months).

### 2.5 Statistical analysis

Normally distributed variables were summarized as mean ± SD and compared across groups using one-way ANOVA, whereas skewed variables were presented as median (IQR) and analyzed using the Kruskal–Wallis test. Categorical data were expressed as numbers (percentages) and compared using the chi-square test or Fisher’s exact test (21). Missing observations were imputed using multiple imputation with the mice package (22), and details of missingness are provided in **Supplementary Table 1**. Kaplan–Meier survival estimates and log-rank tests were computed using the survival package, and curves were visualized with the survminer package. Cox proportional hazards models were fitted using the survival package to assess the association between HGI and all-cause mortality among patients with NSTEMI who underwent PCI. Covariates for multivariable adjustment were selected from baseline variables showing significant differences between patients with and without all-cause death (**Supplementary Table 2**), together with clinically relevant factors identified from prior studies and clinical knowledge (23, 24). Three progressively adjusted Cox proportional hazards models were constructed: Model 1 was unadjusted; Model 2 included variables selected from the between-group comparison; and Model 3 further included established prognostic factors for coronary artery disease. Restricted cubic spline (RCS) analysis with three knots was performed using the rms package to explore potential nonlinear associations. Multicollinearity among covariates included in the multivariable models was assessed using the car package by calculating variance inflation factors (VIFs). A VIF > 5 was considered indicative of significant collinearity. The VIF values of the included covariates are presented in **Supplementary Table 3**. Subgroup analyses were additionally performed to assess the consistency of the findings across clinically relevant categories. Statistical analyses were conducted in R (version 4.4.0), and P-values < 0.05 were considered statistically significant.

## 3 Results

### 3.1 Baseline characteristics

A total of 635 patients were included and stratified into HGI tertiles: T1 (HGI < −0.42, n = 212), T2 (−0.42 ≤ HGI < 0.10, n = 211), and T3 (HGI ≥ 0.10, n = 212). Baseline characteristics are shown in **Table 1**. Age differed significantly across tertiles (P=0.040). Regarding metabolic variables, diabetes prevalence was significantly higher in T3 than in T1 and T2 (P < 0.001), and both FBG and HbA1c reached their highest levels in T3 (both P < 0.001). Significant differences were observed for TG, HDL-C, and LDL-C. Specifically, the T3 group showed the highest TG and lowest HDL-C levels, whereas LDL-C was higher in the T2 group (P < 0.001, P < 0.001, and P=0.027, respectively). All-cause mortality also varied across HGI tertiles (P=0.048), with the highest rate in T1 (9.0%) and the lowest in T3 (3.3%). No significant differences were found between the three groups in the other baseline characteristics, including sex, BMI, blood pressure, smoking and drinking history, history of hypertension, Killip class, previous PCI history, discharge medications, coronary lesion characteristics, or other laboratory parameters (all P > 0.05). In addition, we compared baseline characteristics between patients with and without all-cause death; the relevant results are presented in **Supplementary Table 2**.

**Table 1:** Baseline characteristics according to HGI tertiles.

Characteristics	Overall N = 635	T1 (N= 212) HGI<-0.42	T2 (N= 211) -0.42≤HGI<0.10	T3 (N= 212) HGI≥0.10	P-value
**AGE**	62.00 (55.00, 69.00)	61.00 (53.00, 68.00)	63.00 (57.00, 69.00)	63.00 (56.00, 69.00)	**0.040**
**GENDER**					0.9
NO	128 (20%)	41 (19%)	42 (20%)	45 (21%)	
YES	507 (80%)	171 (81%)	169 (80%)	167 (79%)	
**BMI**	25.95 (24.03, 28.52)	25.61 (24.05, 28.40)	25.71 (23.62, 28.28)	26.67 (24.55, 28.88)	0.081
**SBP**	134.00 (123.00, 146.00)	133.00 (122.50, 148.00)	133.00 (121.00, 147.00)	135.50 (123.00, 145.00)	0.7
**DBP**	75.00 (67.00, 83.00)	76.00 (66.00, 84.00)	75.00 (67.00, 82.00)	74.50 (67.00, 82.50)	0.8
**Smoke**					0.4
NO	390 (61%)	127 (60%)	125 (59%)	138 (65%)	
YES	245 (39%)	85 (40%)	86 (41%)	74 (35%)	
**Drink**					0.4
NO	289 (46%)	95 (45%)	90 (43%)	104 (49%)	
YES	346 (54%)	117 (55%)	121 (57%)	108 (51%)	
**Hypertension**					0.2
NO	231 (36%)	79 (37%)	85 (40%)	67 (32%)	
YES	404 (64%)	133 (63%)	126 (60%)	145 (68%)	
**Diabetes**					**<0.001**
**NO**	359 (57%)	151 (71%)	157 (74%)	51 (24%)	
**YES**	276 (43%)	61 (29%)	54 (26%)	161 (76%)	
**Killip class**					0.14
1	472 (74%)	170 (80%)	148 (70%)	154 (73%)	
2	125 (20%)	33 (16%)	45 (21%)	47 (22%)	
3	36 (5.7%)	9 (4.2%)	17 (8.1%)	10 (4.7%)	
4	2 (0.3%)	0 (0%)	1 (0.5%)	1 (0.5%)	
**Prior PCI**					0.12
NO	504 (79%)	176 (83%)	169 (80%)	159 (75%)	
YES	131 (21%)	36 (17%)	42 (20%)	53 (25%)	
**Aspirin**					0.086
NO	14 (2.2%)	6 (2.8%)	7 (3.3%)	1 (0.5%)	
YES	621 (98%)	206 (97%)	204 (97%)	211 (100%)	
**Clopidogrel**					0.6
NO	59 (9.3%)	16 (7.5%)	21 (10.0%)	22 (10%)	
YES	576 (91%)	196 (92%)	190 (90%)	190 (90%)	
**ACEI/ARB**					0.6
NO	139 (22%)	51 (24%)	43 (20%)	45 (21%)	
YES	496 (78%)	161 (76%)	168 (80%)	167 (79%)	
**β blocker**					0.10
NO	197 (31%)	67 (32%)	75 (36%)	55 (26%)	
YES	438 (69%)	145 (68%)	136 (64%)	157 (74%)	
**Statin**					>0.9
**NO**	582 (91.7%)	195 (92%)	194 (92%)	193 (91%)	
**YES**	53 (8.3%)	17 (8.0%)	17 (8.1%)	19 (9.0%)	
**LM disease**					0.9
NO	614 (97%)	205 (97%)	205 (97%)	204 (96%)	
YES	21 (3.3%)	7 (3.3%)	6 (2.8%)	8 (3.8%)	
**Three vessel disease**					0.8
NO	579 (91%)	191 (90%)	194 (92%)	194 (92%)	
YES	56 (8.8%)	21 (9.9%)	17 (8.1%)	18 (8.5%)	
**ALT**	20.00 (14.00, 27.00)	21.00 (15.00, 28.00)	19.00 (14.00, 24.00)	20.00 (14.00, 29.00)	0.053
**AST**	28.00 (21.00, 44.00)	29.00 (20.50, 54.00)	29.00 (21.00, 41.00)	27.50 (21.00, 41.50)	0.4
**eGFR**	98.49 (87.01, 105.48)	99.17 (86.03, 105.44)	97.49 (87.17, 105.24)	98.56 (87.38, 106.43)	0.9
**BUN**	5.26 (4.22, 6.68)	5.17 (4.26, 6.40)	5.34 (4.31, 6.62)	5.38 (4.14, 6.91)	0.8
**UA**	347.00 (290.00, 416.00)	354.00 (301.00, 426.00)	354.00 (301.00, 414.00)	331.50 (272.00, 396.00)	0.053
**HDL**	0.95 (0.81, 1.13)	0.98 (0.82, 1.14)	0.97 (0.85, 1.17)	0.89 (0.77, 1.06)	**<0.001**
**LDL**	2.55 (1.99, 3.12)	2.61 (2.08, 3.09)	2.74 (2.05, 3.32)	2.40 (1.90, 3.05)	**0.027**
**TG**	1.62 (1.22, 2.36)	1.56 (1.15, 2.26)	1.57 (1.12, 2.17)	1.76 (1.34, 2.75)	**<0.001**
**TC**	4.26 (3.54, 4.90)	4.25 (3.63, 4.81)	4.42 (3.61, 4.98)	4.08 (3.45, 4.89)	0.2
**FBG**	6.03 (5.07, 8.05)	5.71 (5.08, 7.99)	5.40 (4.84, 6.74)	7.44 (5.82, 8.95)	**<0.001**
**HBA1C**	6.20 (5.60, 7.50)	5.60 (5.30, 6.10)	5.90 (5.70, 6.40)	7.70 (6.90, 9.50)	**<0.001**
**WBC**	7.99 (6.56, 9.26)	7.77 (6.41, 9.23)	8.01 (6.74, 9.19)	8.09 (6.57, 9.29)	0.7
**Hemoglobin**	136.31 (16.14)	137.53 (15.78)	136.64 (15.45)	134.77 (17.08)	0.2
**PLT**	208.00 (174.00, 248.00)	208.00 (172.00, 248.50)	211.00 (176.00, 243.00)	204.00 (172.00, 254.00)	>0.9
**LVEF**	61.00 (55.00, 66.00)	61.00 (55.00, 67.00)	60.00 (55.00, 66.00)	60.00 (54.00, 65.50)	0.2
**HGI**	-0.21 (-0.57, 0.39)	-0.75 (-1.06, -0.57)	-0.21 (-0.31, -0.08)	0.84 (0.39, 1.63)	**<0.001**
**All-cause mortality**					**0.048**
NO	597 (94%)	193 (91%)	199 (94%)	205 (97%)	
YES	38 (6.0%)	19 (9.0%)	12 (5.7%)	7 (3.3%)	

BMI, body mass index; SBP, systolic blood pressure; UA, uric acid; eGFR, estimated glomerular filtration rate; ALT, alanine aminotransferase; AST, aspartate aminotransferase; DBP, diastolic blood pressure; HDL, high-density lipoprotein; LDL, low-density lipoprotein; TG, triglycerides; TC, total cholesterol; FBG, fasting blood glucose; HbA1c, glycated hemoglobin; WBC, white blood cell count; PLT, platelet count; HGI, hemoglobin glycation index.

Bold numbers indicate P < 0.05.

### 3.2 Survival analysis

**Figure 3:**
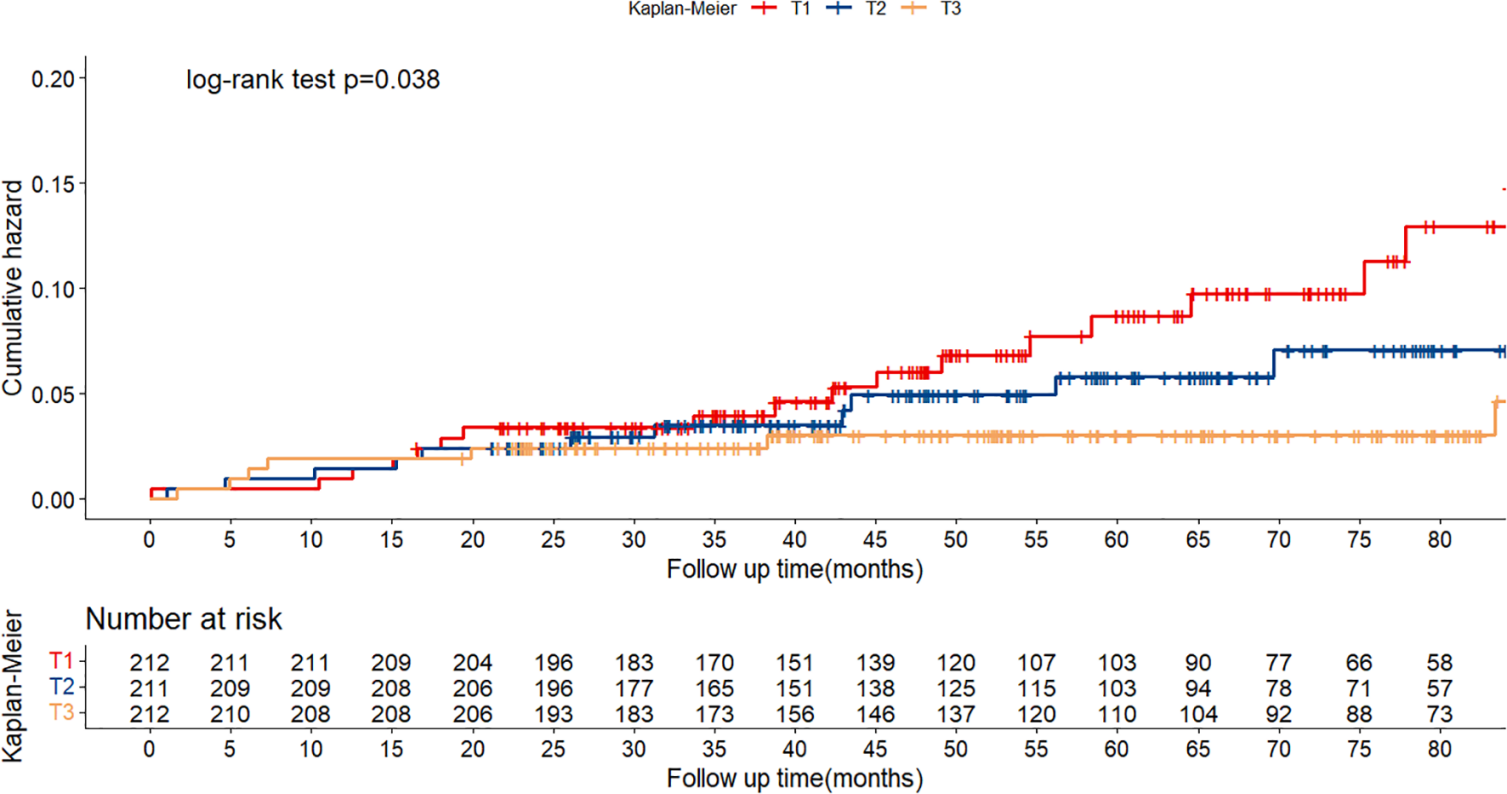
Kaplan–Meier curves of all-cause mortality stratified by HGI tertiles.

Kaplan–Meier curves revealed a significant difference in all-cause mortality among HGI tertiles. The cumulative mortality rate was highest in the T1 group and lower in the T2 and T3 groups. Log-rank testing showed a significant difference across groups (P=0.038), supporting an inverse relationship between HGI and all-cause mortality (**Figure 3**).

### 3.3 Association between HGI and all-cause mortality

**Figure 4:**
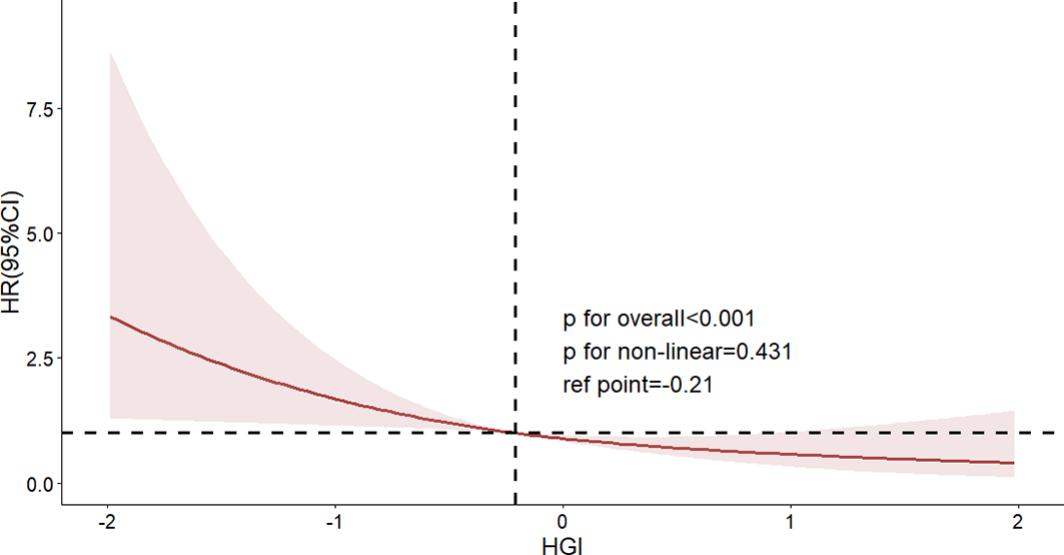
RCS curve for the association between HGI and all-cause mortality.

Treating HGI as a continuous variable yielded a consistent inverse relationship with all-cause mortality across all Cox models (**Table 2**). Without covariate adjustment, a 1-unit increase in HGI was associated with a 44% reduction in mortality risk (HR=0.56, 95% CI: 0.40–0.77, P < 0.001). This association remained significant after adjustment for age, BMI, ALT, eGFR, LDL-C, TC, FBG, and hemoglobin in Model 2 (HR=0.59, 95% CI: 0.42–0.84, P=0.003), and was largely unchanged after further inclusion of sex, hypertension, diabetes, smoking, and drinking status in Model 3 (HR=0.57, 95% CI: 0.40–0.82, P=0.002). A tertile-based analysis produced similar results. Using T1 as the comparator, T3 was associated with lower all-cause mortality in Model 1 (HR=0.35, 95% CI: 0.15–0.84, P=0.018), while T2 showed no statistically significant difference from T1 (HR=0.58, 95% CI: 0.28–1.22, P=0.151). This overall pattern remained after covariate adjustment. In Model 2, HRs were 0.59 (95% CI: 0.27–1.29, P=0.187) for T2 and 0.28 (95% CI: 0.11–0.67, P=0.004) for T3. In Model 3, the respective values were 0.61 (95% CI: 0.28–1.32, P=0.208) and 0.26 (95% CI: 0.10–0.66, P=0.004). RCS analysis was applied to explore whether the association between HGI and all-cause mortality deviated from linearity. No evidence of a nonlinear relationship was observed (**Figure 4**).

**Table 2:** Association between HGI and all-cause mortality.

Variables	Model 1			Model 2			Model 3		
	HR	95%CI	P-value	HR	95%CI	P-value	HR	95%CI	P-value
HGI	0.56	0.40-0.77	<0.001	0.59	0.42-0.84	0.003	0.57	0.40-0.82	0.002
HGI tertile									
T_1_	Ref			Ref			Ref		
T_2_	0.58	0.28-1.22	0.151	0.59	0.27-1.29	0.187	0.61	0.28-1.32	0.208
T_3_	0.35	0.15-0.84	0.018	0.28	0.11-0.67	0.004	0.26	0.10-0.66	0.004
P for trend			0.014			0.003			0.003

Model 1: unadjusted.

Model 2: adjusted for Age, BMI, ALT, eGFR, LDL, TC, FBG, Hemoglobin.

Model 3: adjusted for Age, Gender, BMI, hypertension, diabetes, smoke, drink, ALT, eGFR, LDL, TC, FBG, Hemoglobin.

### 3.4 Subgroup analysis

**Figure 5:**
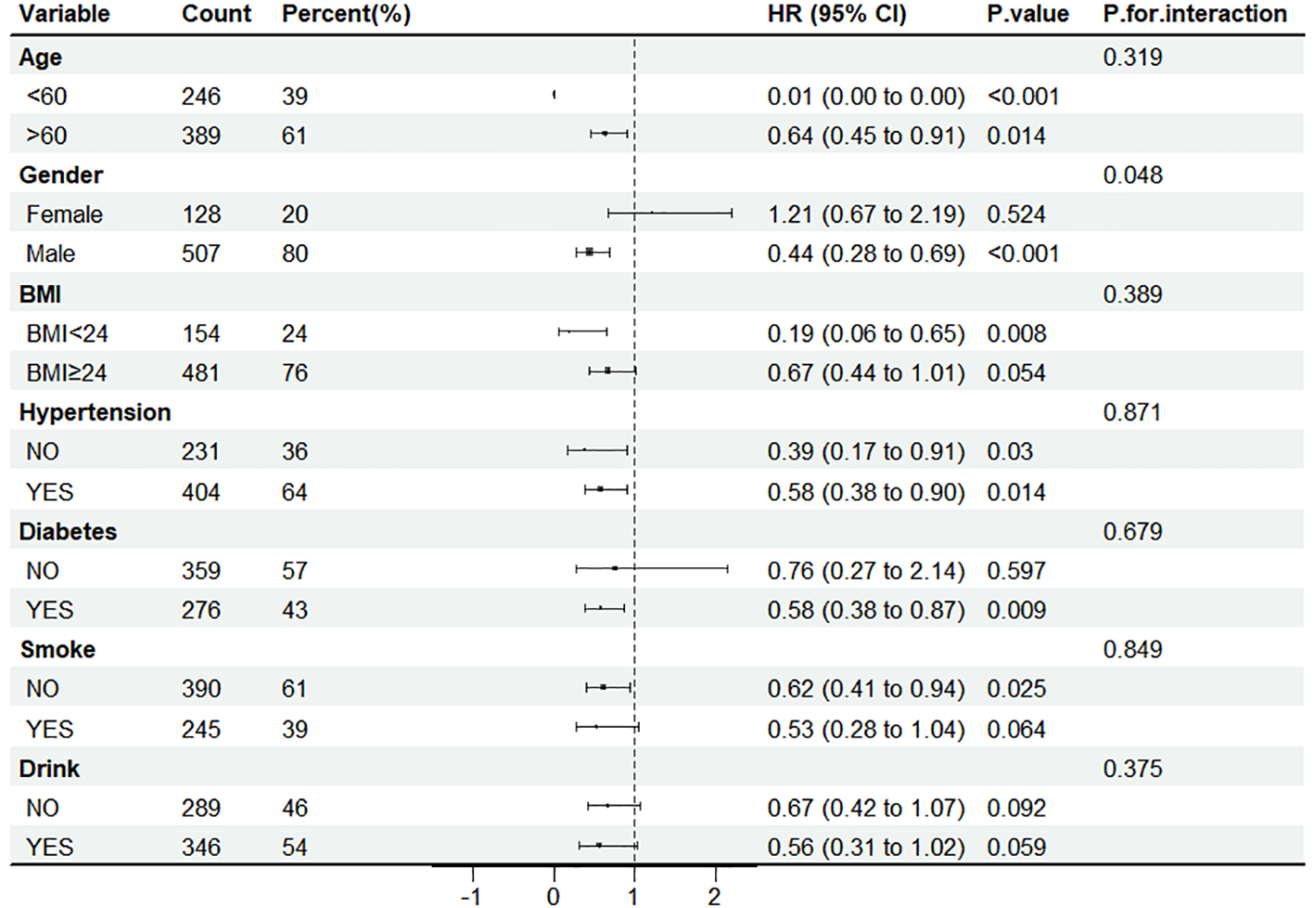
Forest plot illustrating subgroup-specific associations of HGI with all-cause mortality.

Prespecified subgroup analyses were performed to assess the consistency of the association between HGI and all-cause mortality across subgroups. Exploratory subgroup analysis suggested a possible sex-related interaction (P for interaction = 0.048), with the inverse association appearing more evident in men; however, this finding should be interpreted cautiously given the limited sample size. No significant effect modification was identified for age, BMI, smoking status, drinking status, hypertension, or diabetes, indicating a generally consistent association across these strata (**Figure 5**).

### 3.5 Sensitivity analyses

Sensitivity analyses were performed using alternative adjustment strategies to minimize potential overadjustment. Specifically, models excluding FBG and diabetes history were constructed, and the association between HGI and all-cause mortality remained virtually unchanged (**Supplementary Table 4**).

## 4 Discussion

In this cohort of patients with NSTEMI undergoing PCI, we found that HGI was inversely associated with the risk of all-cause mortality. RCS analysis indicated a linear association between HGI and mortality risk. Exploratory subgroup analyses suggested a potential interaction by sex, which should be interpreted cautiously given the limited sample size.

The association between HGI and mortality may vary according to whether patients are in a chronic stable condition or an acute stress state. In relatively stable populations, a higher HGI was associated with an increased risk of all-cause mortality. For example, He et al., in a community-based cohort of approximately 4,800 participants, reported that elevated HGI was associated with higher all-cause mortality (25). Similarly, Huang et al., using data from NHANES, demonstrated that higher HGI was significantly associated with all-cause mortality among patients with diabetic kidney disease (26). In contrast, in certain acutely ill or critically ill populations, higher HGI showed an apparently protective association with all-cause mortality. Yang et al. reported that higher HGI levels were associated with lower all-cause mortality among ICU patients with chronic kidney disease (27). In patients with AMI, several studies based on the MIMIC database also reported an inverse association between HGI and mortality (14, 28). Likewise, our analysis of patients with NSTEMI undergoing PCI showed that higher HGI was associated with a lower risk of all-cause mortality, which is consistent with these previous findings.

Several biological mechanisms may underlie the seemingly conflicting associations between HGI and mortality across different clinical settings. In relatively stable chronic disease populations, a higher HGI reflects higher-than-expected HbA1c at a given glucose level, suggesting an increased tendency toward hemoglobin glycation. This enhanced glycation tendency may promote the formation of advanced glycation end products (AGEs) (29), which can trigger oxidative stress, chronic inflammation, and endothelial dysfunction, thereby accelerating atherosclerosis and vascular injury. In addition, a higher HGI may reflect postprandial hyperglycemia that is not captured by FBG. Therefore, in stable populations, a higher HGI may represent both an enhanced glycation phenotype and poorer glycemic control, which together may contribute to adverse long-term outcomes. In contrast, the physiological significance of HGI may be fundamentally different in patients with AMI or in critically ill patients. Acute stress activates the sympathetic nervous system and neurohormonal pathways, resulting in catecholamine release, cortisol release, inflammatory activation, hepatic gluconeogenesis, and transient insulin resistance (30, 31). These responses may rapidly elevate blood glucose levels at hospital admission. However, HbA1c reflects the average glycemic exposure over the preceding 2–3 months and remains relatively unchanged during acute events. Consequently, the predicted HbA1c derived from admission glucose may rise disproportionately relative to the measured HbA1c, leading to lower or even negative HGI values. Under such circumstances, a lower HGI no longer indicates a favorable chronic metabolic status, but instead may reflect stress hyperglycemia and greater acute disease severity. Importantly, the association between stress hyperglycemia and adverse outcomes after AMI has been well established through multiple mechanisms, including endothelial dysfunction, oxidative stress, platelet activation, coronary microvascular injury, larger infarct size, malignant arrhythmias, and adverse ventricular remodeling (32–36). These mechanisms partly explain why, in our cohort of patients with NSTEMI undergoing PCI, lower HGI was associated with higher mortality risk.

Interestingly, studies in patients with AMI have shown that the association between HGI and mortality has not been consistent, but rather has exhibited different nonlinear patterns. Data from the CAMI registry demonstrated that in-hospital mortality gradually decreased with increasing HGI and then stabilized after reaching a certain threshold (37). Some studies based on the MIMIC database in critically ill patients with AMI also observed a similar trend, in which mortality risk decreased as HGI increased and then plateaued (28, 38). However, other studies using the MIMIC database suggested that mortality first decreased with increasing HGI and then increased again at higher HGI levels (14, 39). In contrast, our study observed a linear inverse association between HGI and all-cause mortality among patients with NSTEMI undergoing PCI. These discrepancies may reflect the different clinical meanings of HGI across populations. First, the CAMI registry focused on in-hospital mortality, an outcome more strongly influenced by acute-phase metabolic disturbances. At this stage, lower HGI often indicates marked stress hyperglycemia, whereas higher HGI may reflect relative hypoglycemia, intensive glucose-lowering therapy, or increased glycemic variability. Therefore, extreme HGI levels at both ends may increase the risk of in-hospital mortality. Second, studies based on the MIMIC database mainly included ICU patients with AMI. These patients were more severely ill and often had shock, infection, and organ dysfunction, resulting in a more complex metabolic state and a greater likelihood of diverse nonlinear associations between HGI and mortality. In contrast, our study focused on patients with NSTEMI undergoing PCI, who represented a relatively more homogeneous population, and primarily evaluated long-term outcomes; therefore, the association pattern may differ from that observed in critically ill patients during the acute phase. In addition, previous studies have reported a linear association between stress hyperglycemia and all-cause mortality, which may partly explain the persistent inverse association observed in our study. Finally, the relatively limited sample size and number of outcome events in our study may also have reduced the statistical power to detect more complex nonlinear relationships.

Several limitations merit consideration. The retrospective nature of the study precludes complete control of residual confounding, despite multivariable adjustment. In addition, the single-center setting and moderate sample size may limit the generalizability of the results, underscoring the need for confirmation in larger multicenter cohorts. Moreover, HGI was calculated using a regression equation derived from the relationship between FBG and HbA1c within the present cohort. Because this relationship may vary according to population characteristics and clinical settings, the resulting HGI values may be population-specific and should be interpreted cautiously when extrapolated to other cohorts. Furthermore, the regression model was based on admission FBG, which in patients with NSTEMI may be partly influenced by acute stress responses and may not fully represent stable chronic glycemic status. Therefore, the calculated HGI may capture both chronic glycemic characteristics and acute-phase metabolic perturbations, thereby further limiting its generalizability.

## 5 Conclusion

Overall, lower HGI was associated with a greater risk of all-cause mortality in patients with NSTEMI undergoing PCI, with the association appearing linear. These findings support the potential complementary role of HGI as an adjunctive marker for prognostic assessment and clinical risk stratification in this population.

## Data Availability

The raw data supporting the conclusions of this article will be made available by the authors, without undue reservation.

## References

[1] Salari N Morddarvanjoghi F Abdolmaleki A Rasoulpoor S Khaleghi AA Hezarkhani LA . The global prevalence of myocardial infarction: a systematic review and meta-analysis. BMC Cardiovasc Disord. (2023) 23:206. doi: 10.1186/s12872-023-03231-w. PMID: 37087452 PMC10122825

[2] Fu R Song C Yang J Wang Y Li B Xu H . CAMI-NSTEMI score - China acute myocardial infarction registry-derived novel tool to predict in-hospital death in non-ST segment elevation myocardial infarction patients. Circ J. (2018) 82:1884–91. doi: 10.1253/circj.cj-17-1078. PMID: 29887577

[3] Damman P van Geloven N Wallentin L Lagerqvist B Fox KA Clayton T . Timing of angiography with a routine invasive strategy and long-term outcomes in non-ST-segment elevation acute coronary syndrome: a collaborative analysis of individual patient data from the FRISC II (Fragmin and Fast Revascularization During Instability in Coronary Artery Disease), ICTUS (Invasive Versus Conservative Treatment in Unstable Coronary Syndromes), and RITA-3 (Intervention Versus Conservative Treatment Strategy in Patients With Unstable Angina or Non-ST Elevation Myocardial Infarction) Trials. JACC Cardiovasc Interventions. (2012) 5:191–9. doi: 10.1016/j.jcin.2011.10.016 22361604

[4] Lagerqvist B Husted S Kontny F Ståhle E Swahn E Wallentin L . 5-year outcomes in the FRISC-II randomised trial of an invasive versus a non-invasive strategy in non-ST-elevation acute coronary syndrome: a follow-up study. Lancet. (2006) 368:998–1004. doi: 10.1016/s0140-6736(06)69416-6. PMID: 16980115

[5] Song Y Chen X Chang Z Bian X He J Li B . The cholesterol, high-density lipoprotein, and glucose (CHG) index as a novel metabolic marker for predicting adverse outcomes in myocardial infarction survivors: insights from two large prospective cohorts. Cardiovasc Diabetol. (2026) 25:80. doi: 10.1186/s12933-026-03104-4. PMID: 41731494 PMC12980928

[6] Marx N Federici M Schütt K Müller-Wieland D Ajjan RA Antunes MJ . 2023 ESC Guidelines for the management of cardiovascular disease in patients with diabetes. Eur Heart J. (2023) 44:4043–140. doi: 10.1093/eurheartj/ehad881. PMID: 37622663

[7] Yu Q Fu Q Ma X Wang H Xia Y Chen Y . Impact of glycemic control metrics on short- and long-term mortality in transcatheter aortic valve replacement patients: a retrospective cohort study from the MIMIC-IV database. Cardiovasc Diabetol. (2025) 24:135. doi: 10.1186/s12933-025-02684-x. PMID: 40121436 PMC11929336

[8] Hempe JM Liu S Myers L McCarter RJ Buse JB Fonseca V . The hemoglobin glycation index identifies subpopulations with harms or benefits from intensive treatment in the ACCORD trial. Diabetes Care. (2015) 38:1067–74. doi: 10.2337/dc14-1844. PMID: 25887355 PMC4439529

[9] Wang S Chen Y Ma H Wang Y Luo M Xie X . Direct Bilirubin, but not Indirect Bilirubin, is Associated with Short-term Adverse Events in HFpEF. Curr Gene Ther. (2024) 24:321–30. doi: 10.2174/0115665232273115240102043640. PMID: 38310459

[10] Hempe JM Gomez R McCarter RJ Chalew SA . High and low hemoglobin glycation phenotypes in type 1 diabetes: a challenge for interpretation of glycemic control. J Diabetes Complications. (2002) 16:313–20. doi: 10.1016/s1056-8727(01)00227-6. PMID: 12200073

[11] Ahn CH Min SH Lee DH Oh TJ Kim KM Moon JH . Hemoglobin glycation index is associated with cardiovascular diseases in people with impaired glucose metabolism. J Clin Endocrinol Metab. (2017) 102:2905–13. doi: 10.1210/jc.2017-00191. PMID: 28541544 PMC6283438

[12] Jia D Jiang M Feng J Pan J Tao Y Wang S . The synergistic predictive value of hemoglobin glycation index and SYNTAX score for coronary artery disease complexity and long-term prognosis after percutaneous coronary intervention. Front Endocrinol (Lausanne). (2025) 16:1727187. doi: 10.3389/fendo.2025.1727187. PMID: 41607456 PMC12834722

[13] Lyu C Tong X Fu P Gao Y Hua J Zhao J . The hemoglobin glycation index stratifies heart failure phenotypes and in-hospital risk. Front Endocrinol (Lausanne). (2025) 16:1740447. doi: 10.3389/fendo.2025.1740447. PMID: 41473250 PMC12745197

[14] Yu L Chen H Zhang J Han W . Relationship between hemoglobin glycation index and all-cause mortality in patients with acute myocardial infarction: based on MIMIC-IV database. Diabetol Metab Syndr. (2025) 17:413. doi: 10.1186/s13098-025-01961-9. PMID: 41174801 PMC12577028

[15] Cao H Gui L Hu Y Yang J Hua P Yang S . Association between hemoglobin glycation index and adverse outcomes in critically ill patients with myocardial infarction: A retrospective cohort study. Nutrition Metab Cardiovasc Dis. (2025) 35:103973. doi: 10.1016/j.numecd.2025.103973. PMID: 40180831

[16] Cui K Fu R Yang J Xu H Wu W Chen K . Haemoglobin glycation index and in‐hospital mortality after acute myocardial infarction in patients with/without diabetes: A prospective, nationwide and multicentre registry. Diabetes Obes Metab. (2025) 27:4511–21. doi: 10.1111/dom.16495. PMID: 40459018

[17] Diabetes* ADAPPCf . Introduction and methodology: standards of care in diabetes—2026. Diabetes Care. (2025) 49:S1–5. doi: 10.2337/dc26-sint. PMID: 41358883 PMC12690168

[18] McEvoy JW McCarthy CP Bruno RM Brouwers S Canavan MD Ceconi C . 2024 ESC Guidelines for the management of elevated blood pressure and hypertension. Eur Heart J. (2024) 45:3912–4018. doi: 10.1093/eurheartj/ehae178. PMID: 39210715

[19] Byrne RA Rossello X Coughlan JJ Barbato E Berry C Chieffo A . 2023 ESC Guidelines for the management of acute coronary syndromes. Eur Heart J. (2023) 44:3720–826. doi: 10.1093/ehjacc/zuad107. PMID: 37622654

[20] Rao SV O’Donoghue ML Ruel M Rab T Tamis-Holland JE Alexander JH . 2025 ACC/AHA/ACEP/NAEMSP/SCAI guideline for the management of patients with acute coronary syndromes: A report of the American college of cardiology/American heart association joint committee on clinical practice guidelines. Circulation. (2025) 151:e771–e862. doi: 10.1161/cir.0000000000001309. PMID: 40014670

[21] Han SS Liu Q Zeng ZM Li Y Li PW Cheng FX . Association of various insulin resistance surrogate indices with aging acceleration and future risk of cardiovascular disease in individuals with cardiovascular-kidney-metabolic syndrome stages 0-3: insights from CHARLS 2011–2020 data. Cardiovasc Diabetol. (2026) 25:77. doi: 10.1186/s12933-026-03084-5. PMID: 41639853 PMC12964732

[22] Zheng W Man Z Ren Y Li Y Zhu X Wang L . Association of the triglyceride glucose-Chinese visceral adiposity index with incident cardiometabolic multimorbidity in middle-aged and older adults: a nationwide prospective cohort study. Cardiovasc Diabetol. (2026) 25:76. doi: 10.1186/s12933-026-03091-6. PMID: 41639722 PMC12964883

[23] Zhang Y Liu B Zhu Y Xie Y Du Y Xiong P . Associations of cumulative exposure and dynamic trajectories of cholesterol-HDL-glucose (CHG) index with cardiovascular disease in middle-aged and older Chinese adults: a longitudinal analysis. Cardiovasc Diabetol. (2026) 25:88. doi: 10.1186/s12933-026-03137-9. PMID: 41832529 PMC12994255

[24] Wang P Yuan D Zhang C Zhu P Jia S Song Y . High fibrinogen-to-albumin ratio with type 2 diabetes mellitus is associated with poor prognosis in patients undergoing percutaneous coronary intervention: 5-year findings from a large cohort. Cardiovasc Diabetol. (2022) 21:46. doi: 10.1093/eurjpc/zwac056.076 35313877 PMC8939137

[25] He J Zhang H Tan Y Guo H Peng H Zheng Y . Nonlinear association of the hemoglobin glycation index with all-cause and cardiovascular mortality: A community-based cohort study. J Diabetes Complications. (2026) 40:109212. doi: 10.1016/j.jdiacomp.2025.109212. PMID: 41218587

[26] Huang L He L Luo X Zhou X . Association of haemoglobin glycation index with all-cause and cardiovascular disease mortality in diabetic kidney disease: a cohort study. Diabetol Metab Syndr. (2024) 16:221. doi: 10.1186/s13098-024-01462-1. PMID: 39261957 PMC11389330

[27] Peng Y Huang W Wang J . Hemoglobin glycation index predicts reduced mortality in critically ill patients with chronic kidney disease. Clinics (Sao Paulo). (2025) 80:100812. doi: 10.1016/j.clinsp.2025.100812. PMID: 41166859 PMC12613056

[28] Chen D Hu B Chen XH Wei X Feng J Hu ZP . Association between different hemoglobin glycation index and prognosis in patients with a first diagnosis of acute myocardial infarction: a retrospective study based on the MIMIC-IV database. Front Cardiovasc Med. (2025) 12:1447420. doi: 10.3389/fcvm.2025.1447420. PMID: 40491719 PMC12146337

[29] Geng L Diao X Yu HJ Xu A . Association of hemoglobin glycation index with the severity and mortality of cardiovascular-kidney-metabolic syndrome: a population-based cohort study. Cardiovasc Diabetol Endocrinol Rep. (2025) 11:46. doi: 10.1186/s40842-025-00262-4. PMID: 41419979 PMC12717684

[30] Sia CH Chan MH Zheng H Ko J Ho AF Chong J . Optimal glucose, HbA1c, glucose-HbA1c ratio and stress-hyperglycaemia ratio cut-off values for predicting 1-year mortality in diabetic and non-diabetic acute myocardial infarction patients. Cardiovasc Diabetol. (2021) 20:211. doi: 10.1186/s12933-021-01395-3. PMID: 34666746 PMC8524932

[31] Paolisso P Bergamaschi L Rambaldi P Gatta G Foà A Angeli F . Impact of admission hyperglycemia on heart failure events and mortality in patients with takotsubo syndrome at long-term follow-up: data from HIGH-GLUCOTAKO investigators. Diabetes Care. (2021) 44:2158–61. doi: 10.2337/dc21-0433. PMID: 34187841

[32] Beverly JK Budoff MJ . Atherosclerosis: Pathophysiology of insulin resistance, hyperglycemia, hyperlipidemia, and inflammation. J Diabetes. (2020) 12:102–4. doi: 10.1111/1753-0407.12970. PMID: 31411812

[33] Wu S Lu Q Ding Y Wu Y Qiu Y Wang P . Hyperglycemia-driven inhibition of AMP-activated protein kinase α2 induces diabetic cardiomyopathy by promoting mitochondria-associated endoplasmic reticulum membranes *in vivo*. Circulation. (2019) 139:1913–36. doi: 10.1161/circulationaha.118.033552. PMID: 30646747 PMC6465113

[34] Song G Liu X Lu Z Guan J Chen X Li Y . Relationship between stress hyperglycaemic ratio (SHR) and critical illness: a systematic review. Cardiovasc Diabetol. (2025) 24:188. doi: 10.1186/s12933-025-02751-3. PMID: 40317019 PMC12049067

[35] Jensen CJ Eberle HC Nassenstein K Schlosser T Farazandeh M Naber CK . Impact of hyperglycemia at admission in patients with acute ST-segment elevation myocardial infarction as assessed by contrast-enhanced MRI. Clin Res Cardiol. (2011) 100:649–59. doi: 10.1007/s00392-011-0290-7. PMID: 21347741

[36] Worthley MI Holmes AS Willoughby SR Kucia AM Heresztyn T Stewart S . The deleterious effects of hyperglycemia on platelet function in diabetic patients with acute coronary syndromes mediation by superoxide production, resolution with intensive insulin administration. J Am Coll Cardiol. (2007) 49:304–10. doi: 10.1016/j.jacc.2006.08.053. PMID: 17239711

[37] Cui K Fu R Yang J Xu H Wu W Chen K . Haemoglobin glycation index and in-hospital mortality after acute myocardial infarction in patients with/without diabetes: A prospective, nationwide and multicentre registry. Diabetes Obes Metab. (2025) 27:4511–21. doi: 10.1111/dom.16495. PMID: 40459018

[38] Lv Y Wei L Wang Z Mu Z Wu J . Association between hemoglobin glycation index and 28-day all-cause mortality in acute myocardial infarction patients: Analysis of the MIMIC-IV database. PLoS One. (2025) 20:e0330819. doi: 10.1371/journal.pone.0330819. PMID: 40892746 PMC12404404

[39] Wei X Chen X Zhang Z Wei J Hu B Long N . Risk analysis of the association between different hemoglobin glycation index and poor prognosis in critical patients with coronary heart disease-A study based on the MIMIC-IV database. Cardiovasc Diabetol. (2024) 23:113. doi: 10.1186/s12933-024-02206-1. PMID: 38555454 PMC10981833

