# Supplementary material for: Association between hemoglobin glycation index and all-cause mortality in patients with non–ST-segment elevation myocardial infarction undergoing percutaneous coronary intervention
Source: Front Endocrinol (Lausanne). 2026 May 25;17:1847325. doi: 10.3389/fendo.2026.1847325 (PMC13243125; doi:10.3389/fendo.2026.1847325)
Supplement: Supplementary file 1 [file Supplementaryfile1.docx]

**Table S1 Proportion of missing values for study variables**

| Variables | Missing number (%) |
| --- | --- |
| BMI | 6% |
| SBP | 0.3% |
| DBP | 0.5% |
| AGE | 1.4% |
| HDL | 0.3% |
| LDL | 0.2% |
| WBC | 0.3% |
| Hemoglobin | 0.6% |
| LVEF | 4.9% |
| Aspirin | 14.2% |
| Clopidogrel | 14.5% |
| ACEI/ARB | 14.5% |
| β blocker | 14.5% |
| Statin | 14.5% |

Abbreviation: body mass index: BMI; systolic blood pressure: SBP; diastolic blood pressure: DBP; high-density lipoprotein: HDL; low-density lipoprotein: LDL, white blood cell count: WBC;

**Table S2 Baseline characteristics according to all-cause mortality status**

| **Characteristic** | **Overall**  N = 635 | **No all-cause death**  N = 597 | **All-cause death** N = 38 | **p-value** |
| --- | --- | --- | --- | --- |
| **AGE** | 62.00 (55.00, 69.00) | 62.00 (55.00, 68.00) | 72.00 (64.00, 76.00) | **<0.001** |
| **GENDER** |  |  |  | 0.6 |
| Female | 128 (20%) | 119 (20%) | 9 (24%) |  |
| Male | 507 (80%) | 478 (80%) | 29 (76%) |  |
| **BMI** | 25.95 (24.03, 28.52) | 26.12 (24.06, 28.58) | 25.01 (23.57, 26.67) | **0.028** |
| **SBP** | 134.00 (123.00, 146.00) | 134.00 (123.00, 146.00) | 136.00 (123.00, 152.00) | 0.4 |
| **DBP** | 75.00 (67.00, 83.00) | 75.00 (67.00, 83.00) | 71.00 (64.00, 80.00) | 0.11 |
| **Smoke** |  |  |  | 0.8 |
| NO | 390 (61%) | 366 (61%) | 24 (63%) |  |
| YES | 245 (39%) | 231 (39%) | 14 (37%) |  |
| **Drink** |  |  |  | 0.4 |
| NO | 289 (46%) | 269 (45%) | 20 (53%) |  |
| YES | 346 (54%) | 328 (55%) | 18 (47%) |  |
| **Hypertension** |  |  |  | 0.5 |
| NO | 231 (36%) | 219 (37%) | 12 (32%) |  |
| YES | 404 (64%) | 378 (63%) | 26 (68%) |  |
| **Diabetes** |  |  |  | 0.2 |
| NO | 359 (57%) | 341 (57%) | 18 (47%) |  |
| YES | 276 (43%) | 256 (43%) | 20 (53%) |  |
| **Killip class** |  |  |  | 0.090 |
| 1 | 472 (74%) | 450 (75%) | 22 (58%) |  |
| 2 | 125 (20%) | 112 (19%) | 13 (34%) |  |
| 3 | 36 (5.7%) | 33 (5.5%) | 3 (7.9%) |  |
| 4 | 2 (0.3%) | 2 (0.3%) | 0 (0%) |  |
| **Prior PCI** |  |  |  | >0.9 |
| NO | 504 (79%) | 474 (79%) | 30 (79%) |  |
| YES | 131 (21%) | 123 (21%) | 8 (21%) |  |
| **Aspirin** |  |  |  | >0.9 |
| NO | 14 (2.2%) | 14 (2.3%) | 0 (0%) |  |
| YES | 621 (98%) | 583 (98%) | 38 (100%) |  |
| **Clopidogrel** |  |  |  | 0.4 |
| NO | 59 (9.3%) | 54 (9.0%) | 5 (13%) |  |
| YES | 576 (91%) | 543 (91%) | 33 (87%) |  |
| **ACEI/ARB** |  |  |  | 0.9 |
| NO | 139 (22%) | 131 (22%) | 8 (21%) |  |
| YES | 496 (78%) | 466 (78%) | 30 (79%) |  |
| **β blocker** |  |  |  | >0.9 |
| NO | 197 (31%) | 185 (31%) | 12 (32%) |  |
| YES | 438 (69%) | 412 (69%) | 26 (68%) |  |
| **Statin** |  |  |  | 0.2 |
| NO | 53 (8.3%) | 48 (8.0%) | 5 (13%) |  |
| YES | 582 (92%) | 549 (92%) | 33 (87%) |  |
| **LM disease** |  |  |  | 0.4 |
| NO | 614 (97%) | 578 (97%) | 36 (95%) |  |
| YES | 21 (3.3%) | 19 (3.2%) | 2 (5.3%) |  |
| **Three vessel disease** |  |  |  | 0.8 |
| NO | 579 (91%) | 545 (91%) | 34 (89%) |  |
| YES | 56 (8.8%) | 52 (8.7%) | 4 (11%) |  |
| **ALT** | 20.00 (14.00, 27.00) | 20.00 (15.00, 28.00) | 13.50 (11.00, 23.00) | **<0.001** |
| **AST** | 28.00 (21.00, 44.00) | 29.00 (21.00, 44.00) | 24.00 (19.00, 41.00) | 0.12 |
| **eGFR** | 98.49 (87.01, 105.48) | 99.12 (87.93, 105.72) | 89.72 (69.35, 95.86) | **<0.001** |
| **BUN** | 5.26 (4.22, 6.68) | 5.25 (4.20, 6.67) | 5.75 (4.52, 7.04) | 0.3 |
| **UA** | 347.00 (290.00, 416.00) | 350.00 (290.00, 416.00) | 334.50 (298.00, 399.00) | 0.5 |
| **HDL** | 0.95 (0.81, 1.13) | 0.94 (0.81, 1.12) | 1.07 (0.85, 1.19) | 0.086 |
| **LDL** | 2.55 (1.99, 3.12) | 2.58 (2.01, 3.13) | 2.25 (1.69, 2.87) | **0.023** |
| **TG** | 1.62 (1.22, 2.36) | 1.68 (1.24, 2.40) | 1.31 (0.89, 1.74) | **0.001** |
| **TC** | 4.26 (3.54, 4.90) | 4.29 (3.58, 4.91) | 3.64 (3.41, 4.39) | **0.019** |
| **FBG** | 6.03 (5.07, 8.05) | 6.00 (5.06, 7.98) | 6.51 (5.50, 10.01) | **0.047** |
| **HBA1C** | 6.20 (5.60, 7.50) | 6.20 (5.60, 7.50) | 6.20 (5.90, 6.90) | 0.7 |
| **WBC** | 7.99 (6.56, 9.26) | 8.01 (6.57, 9.25) | 7.42 (5.92, 9.47) | 0.3 |
| **Hemoglobin** | 136.31 ± 16.14 | 136.97 ± 16.01 | 126.03 ± 14.82 | **<0.001** |
| **PLT** | 208.00 (174.00, 248.00) | 210.00 (175.00, 248.00) | 201.50 (168.00, 238.00) | 0.3 |
| **LVEF** | 61.00 (55.00, 66.00) | 61.00 (55.00, 66.00) | 60.50 (48.00, 67.00) | 0.5 |
| **HGI** | -0.21 (-0.57, 0.39) | -0.19 (-0.55, 0.44) | -0.40 (-0.87, -0.12) | **0.007** |

Abbreviation: body mass index: BMI; systolic blood pressure: SBP; estimated glomerular filtration rate: eGFR; alanine aminotransferase: ALT; aspartate aminotransferase: AST; diastolic blood pressure: DBP; high-density lipoprotein: HDL; low-density lipoprotein: LDL, triglycerides: TG total cholesterol: TC; fasting blood glucose: FBG; glycated hemoglobin: HbA1c; white blood cell count: WBC; platelet count: PLT; hemoglobin glycation index: HGI

**Table S3 Multicollinearity evaluation of for all variables included multivariable model through VIF analysis**

| Variables | VIF |
| --- | --- |
| Age | 1.896 |
| Gender | 1.514 |
| BMI | 1.025 |
| AGE | 1.4 |
| Hypertension | 1.087 |
| Diabetes | 1.928 |
| Smoke | 1.730 |
| Drink | 1.509 |
| ALT | 1.127 |
| eGFR | 1.662 |
| LDL | 4.582 |
| TC | 4.765 |
| FBG | 1.711 |
| Hemoglobin | 1.449 |

Abbreviation: body mass index: BMI; estimated glomerular filtration rate: eGFR; alanine aminotransferase: ALT; low-density lipoprotein: LDL; TG total cholesterol: TC; fasting blood glucose: FBG;

**Table S4 Association between HGI and all-cause mortality**

|  | Model 1 | | | | Model 2 | | | | | Model 3 | | | |
| --- | --- | --- | --- | --- | --- | --- | --- | --- | --- | --- | --- | --- | --- |
|  | HR | 95%CI | P-value | | | HR | 95%CI | P-value | | | HR | 95%CI | P-value |
| HGI | 0.56 | 0.40-0.77 | | <0.001 | | 0.51 | 0.37-0.72 | | <0.001 | | 0.49 | 0.34-0.69 | <0.001 |
| HGI tertile |  |  | |  | |  |  | |  | |  |  |  |
| T_1_ | Ref |  | |  | | Ref |  | |  | | Ref |  |  |
| T_2_ | 0.58 | 0.28-1.22 | | 0.151 | | 0.52 | 0.24-1.10 | | 0.087 | | 0.52 | 0.25-1.12 | 0.094 |
| T_3_ | 0.35 | 0.15-0.84 | | 0.018 | | 0.28 | 0.11-0.67 | | 0.004 | | 0.28 | 0.12-0.68 | 0.005 |
| P for trend |  |  | | 0.014 | |  |  | | 0.003 | |  |  | 0.003 |

Model 1: unadjusted

Model 2: adjusted for Age, BMI, ALT, eGFR, LDL, TC, Hemoglobin

Model 3:adjusted for Age, Gender, BMI, hypertension, smoke, drink, ALT, eGFR, LDL, TC, Hemoglobin
